# MRI Characterization of Paranodal Junction Failure and Related Spinal Cord Changes in Mice

**DOI:** 10.1371/journal.pone.0052904

**Published:** 2012-12-27

**Authors:** Morito Takano, Keigo Hikishima, Kanehiro Fujiyoshi, Shinsuke Shibata, Akimasa Yasuda, Tsunehiko Konomi, Akiko Hayashi, Hiroko Baba, Koichi Honke, Yoshiaki Toyama, Hideyuki Okano, Masaya Nakamura

**Affiliations:** 1 Department of Orthopaedic Surgery, Keio University School of Medicine, Tokyo, Japan; 2 Department of Physiology, Keio University School of Medicine, Tokyo, Japan; 3 Central Institute for Experimental Animals, Kawasaki, Kanagawa, Japan; 4 National Hospital Organization Murayama Medical Center, Tokyo, Japan; 5 Department of Molecular Neurobiology, Tokyo University of Pharmacy and Life Science, Tokyo, Japan; 6 Department of Biochemistry, Kochi University Medical School, Nankoku, Kochi, Japan; Oregon Health & Science University, United States of America

## Abstract

The paranodal junction is a specialized axon-glia contact zone that is important for normal neuronal activity and behavioral locomotor function in the central nervous system (CNS). Histological examination has been the only method for detecting pathological paranodal junction conditions. Recently, diffusion tensor MRI (DTI) has been used to detect microstructural changes in various CNS diseases. This study was conducted to determine whether MRI and DTI could detect structural changes in the paranodal junctions of the spinal cord in cerebroside sulfotransferase knock-out (CST-KO) mice. Here, we showed that high-resolution MRI and DTI characteristics can reflect paranodal junction failure in CST-KO mice. We found significantly lower T1 times and significantly higher T2 times in the spinal cord MRIs of CST-KO mice as compared to wild-type (WT) mice. Spinal cord DTI showed significantly lower axial diffusivity and significantly higher radial diffusivity in CST-KO mice as compared to WT mice. In contrast, the histological differences in the paranodal junctions of WT and CST-KO mice were so subtle that electron microscopy or immunohistological analyses were necessary to detect them. We also measured gait disturbance in the CST-KO mice, and determined the conduction latency by electrophysiology. These findings demonstrate the potential of using MRI and DTI to evaluate white matter disorders that involve paranodal junction failure.

## Introduction

Myelinated axon segments have four distinct regions: the node of Ranvier, the paranode, the juxtaparanode, and the internode [1]. Each region is involved in the activity and protection of the axon. The paranodal junction, which is a specialized axon-glia contact zone, seals the internodal periaxonal space from the outside milieu. The zone's molecular architecture is built on axonal and glial adhesion proteins linked by scaffolding proteins [2]. Honke et al reported that mice with a disrupted cerebroside sulfotransferase (CST) gene have abnormal or nonfunctional paranodal junctions in the central and peripheral nervous systems [3]–[5]. The CST enzyme synthesizes sulfatide, a major lipid component of the myelin sheath [6], [7]. Significantly abnormal sulfatide levels are found in patients with disorders such as metachromatic leukodystrophy and Alzheimer's disease [8]. Therefore, paranodal junctions and the sulfatide lipid may be involved in the pathogenesis of these neurological disorders.

Diffusion tensor MRI (DTI), a tool that has proven useful in the diagnosis of central nervous disorders [9]–[12], has recently been used to characterize tissue structures at the microscopic level. DTI is particularly helpful in understanding the pathology of neurodegenerative disorders and injuries [13]–[15]. Although transgenic mice are often used to analyze the pathology of white-matter disorders, they are rarely used for *in vivo* MRI of the spinal cord, because their body movement and small size make it difficult to delineate the spinal cord. To overcome these problems, we applied a cryogenic quadrature radio-frequency (RF) surface probe to the mouse spinal cord to improve the sensitivity of the *in vivo* MRI. This cryogenic device has been used successfully to improve the MR imaging of small animals [16].

Although *in vivo* DTI is more difficult to perform due to the longer exposure time required, Sun et al reported that the diffusion anisotropy of white matter is preserved in fixed tissue [17], because DTI information is mainly dominated by static anatomical structures. In this study, we performed *in vivo* and *ex vivo* MRI, including DTI, of the spinal cord of CST-KO mice to determine whether quantitative MRI could be used successfully to evaluate paranodal junction failure in these mice. We compared the MRI findings with histological findings, motor performance, and electrophysiological results.

## Materials and Methods

### Animal model

We used 8-week-old CST-KO mice and, as a control, their wild-type (WT) C57BL6 littermates. Pups obtained from intercrossed heterozygous mice were genotyped by polymerase chain reaction using specific primer sets [3]. WT and CST-KO mice were housed in groups with food and water *ad libitum*, and were maintained under a 12-hour light/dark cycle.

### Ethics statement

All interventions and animal care procedures were performed in accordance with the Laboratory Animal Welfare Act, the Guide for the Care and Use of Laboratory Animals (National Institutes of Health, USA), and the Guidelines and Policies for Animal Surgery provided by the Animal Study Committee of the Central Institute for Experimental Animals and Keio University and were approved by the Animal Study Committee of Keio University (IRB approval number 09091-8).

### Magnetic resonance imaging

MRI was performed with a 7.0-tesla magnet (BioSpec 70/16; Bruker BioSpin, Ettlingen, Germany) and a cryogenic quadrature RF surface probe (CryoProbe; Bruker BioSpin AG, Fällanden, Switzerland) to improve the sensitivity [16], [18]. The cryoprobe technology can lower only the noise of the measurements; it does not affect the contribution of areas outside the paranodal junctions to the MR signal. T1 and T2 MRI scans were performed under general anesthesia induced by intramuscular ketamine (50 mg/kg; Sankyo, Tokyo, Japan) and xylazine (5 mg/kg; Bayer, Leverkusen, Germany) injection, and maintained by isoflurane (Foren; Abbott, Tokyo, Japan). The animal's pulse, arterial oxygen saturation, and rectal temperature were monitored during MRI. For *ex vivo* studies, the animals were euthanized by deep anesthesia (intravenous sodium pentobarbital, 100 mg/kg), and the spinal cord was removed and immersed in 4% paraformaldehyde (PFA) in 0.01 M phosphate-buffered saline (PBS) for 2 weeks. After fixation, the specimens were stored in PBS containing the contrast agent gadopentetate dimeglumine (1 mM; Magnevist, Schering, Berlin, Germany) for 2 weeks. The specimens were then embedded in 2% agarose gel and immediately subjected to MRI.

*In vivo* high-resolution T1 mapping was conducted using rapid acquisition with relaxation enhancement (RARE) and the following parameters: echo time (TE), 18 ms; variable repetition time (TR), 200, 350, 500, 744, 1032, 1384, 2468, 3527, and 8000 ms; RARE factor, 4; number of averages (NA), 4. T2 mapping was conducted using multiple spin-echo with the following parameters: TE, 9, 18, 27, 37, 46, 55, 64, 73, 82, 91, 101, and 110 ms; TR, 3000 ms; RARE factor, 1; NA, 1. The T1- and T2-mapping spatial resolution was 80 µm in-plane and 1.0 mm in thickness. For T2-weighted imaging (T2WI), we used RARE with the following parameters: TE, 31 ms; TR, 3000 ms; RARE factor, 8; NA, 4; spatial resolution, 60 µm in the plane and 1.0 mm in thickness. For both the WT and CST-KO mice, we selected an ROI size sufficient to cover the ventral white matter. The ROI we used was elliptical, with an area of 0.144 mm^2^.

*Ex vivo* DTI data sets were acquired with a spin-echo sequence based on the Stejskal-Tanner diffusion preparation [19], with the following parameters: TE/TR 22.3 ms/1500 ms; *b*-value 1000 sec/mm^2^; motion-probing gradient (MPG), 12 axes; MPG duration, 3 ms; MPG separation, 16 ms. Diffusion tensor images were computed with Diffusion Toolkit software (Martinos Center for Biomedical Imaging, Massachusetts General Hospital; http://www.trackvis.org). We obtained eigenvalues (λ1∼3) by analyzing the diffusion ellipsoid model. We used λ1 as the axial diffusivity (AD), that is, as the diffusivity parallel to fiber orientation in the white matter, and (λ2+λ3)/2 as the radial diffusivity, that is, as the diffusivity perpendicular to the fiber orientation.

### Histological analysis

Eight-week-old WT and CST-KO mice were deeply anesthetized in a humane manner with ketamine (50 mg/kg; Sankyo, Tokyo, Japan) and xylazine (5 mg/kg; Bayer, Leverkusen, Germany) by i.p. injection, then transcardially perfused with 4% PFA in 0.01 M PBS. The spinal cord was removed, embedded in OCT compound (Sakura Finetechnical Co., Ltd., Tokyo, Japan), and sectioned in the sagittal/axial plane at 20 µm on a cryostat (Leica CM3050 S, Wetzlar, Germany). The spinal cord sections were histologically evaluated by immunohistochemistry or hematoxylin-eosin (HE), Luxol Fast Blue (LFB), or Eriochrome Cyanine (EC) staining. To examine the paranodal junctions, sections were stained with the following primary antibodies: anti-Nav1.6 (rabbit, 1∶200, Alomone Labs, Jerusalem, Israel), anti-Caspr (mouse IgG_1_, 1∶200, Neuromab, CA, USA), and anti-Kv1.2 (mouse IgG_2b_, 1∶200, Neuromab, CA, USA). Images were quantified using an inverted fluorescence microscope (BZ 9000; Keyence Co., Osaka, Japan) or a confocal laser-scanning microscope (LSM 700, Carl Zeiss, Munich, Germany). Constant threshold values were maintained for all analyses.

For toluidine blue staining, the samples were fixed in 2.5% glutaraldehyde/0.1 M cacodylate buffer (pH 7.4) for 12 hours, washed in cacodylate buffer, post-fixed for 2 hours in 1% OsO4/0.1 M cacodylate buffer (pH 7.4), dehydrated in a graded alcohol series with acetone, and embedded in epoxy resin. Semi-thin WT and CST-KO spinal cord sections (0.2-µm thick) were stained with toluidine blue (1%) for 20 minutes and examined under a light microscope (BZ 9000; Keyence Co., Osaka, Japan). The axon density was defined as axonal area/total area (200×100 µm^2^).

### Electron microscopy

WT and CST-KO mice were perfused with 4% PFA in 0.01 M PBS at pH 7.4. The spinal cord was dissected and post-fixed with 2.5% glutaraldehyde in 60 mM HEPES (pH 7.4) at 4°C overnight. The samples were fixed for 2 hours in 0.5% osmium tetroxide, dehydrated through ethanol, acetone, and QY1, and embedded in Epon. Ultrathin (80 nm) sagittal spinal cord sections were stained with uranyl acetate and lead citrate for 10 and 12 minutes, respectively. The sections were examined under a transmission electron microscope (JEOL model 1230) and photographed using a Digital Micrograph 3.3 (Gatan Inc., CA, USA).

### Behavioral analyses

A Rotarod treadmill apparatus (Muromachi Kikai Co., Ltd., Tokyo, Japan) and a DigiGait Image Analysis System (Mouse Specifics, Quincy, MA, USA) were used to evaluate motor function in 8-week-old WT and CST-KO mice. In the Rotarod treadmill test, we measured the time (latency) mice spent on a rod rotating at 10 rpm for 2 minutes [20] and recorded the average from three trials. The DigiGait system was used for footprint analysis, in which the forelimb and hindlimb step widths (defined as the distance between the right and left footprint) were measured for as long as the mice could walk with consistent weight-supporting steps on a treadmill set at a speed of 8 cm/s.

### Electrophysiology

Electrophysiological experiments were conducted with an electromyography (EMG)/evoked potential measuring system (Neuropack S1 MEB-9400 series, Nihon Kohden, Tokyo, Japan). WT and CST-KO mice were anesthetized with an i.p. injection of ketamine (40 mg/kg) and xylazine (4 mg/kg), as previously described [21]. An electrode was inserted into the spinal cord at the occipito-cervical area to induce a motor-evoked potential (MEP). The potential was recorded by two needle electrodes, one in each hindlimb; the active electrode was placed in the muscle belly of each limb, and the reference electrode was placed near the distal tendon of the muscle. The ground electrode was placed subcutaneously between the coil and the recording electrodes. To induce MEPs, a 0.4-mA stimulus was applied at the electrode; the pulse duration in all experiments was 0.2 ms. The onset latency was measured as the time in milliseconds between the stimulus and the onset of the first wave. Ten responses were averaged and sorted for off-line analysis [22].

### Statistical Analyses

All values are presented as the mean ± standard deviation (s.d.). After testing for normality, an unpaired two-tailed Student's t-test was used to determine the significance of differences in the MRI findings between the WT and CST-KO groups. The Mann-Whitney test was used to detect significant differences in the histological, behavioral, and MEP findings. For all statistical analyses, significance was defined as p<0.05. GraphPad Prism software (version 5.0d) was used for the analyses (GraphPad Software, Inc., CA, USA).

## Results

### Histological analyses of the WT and CST-KO spinal cord

**Figure 1:**
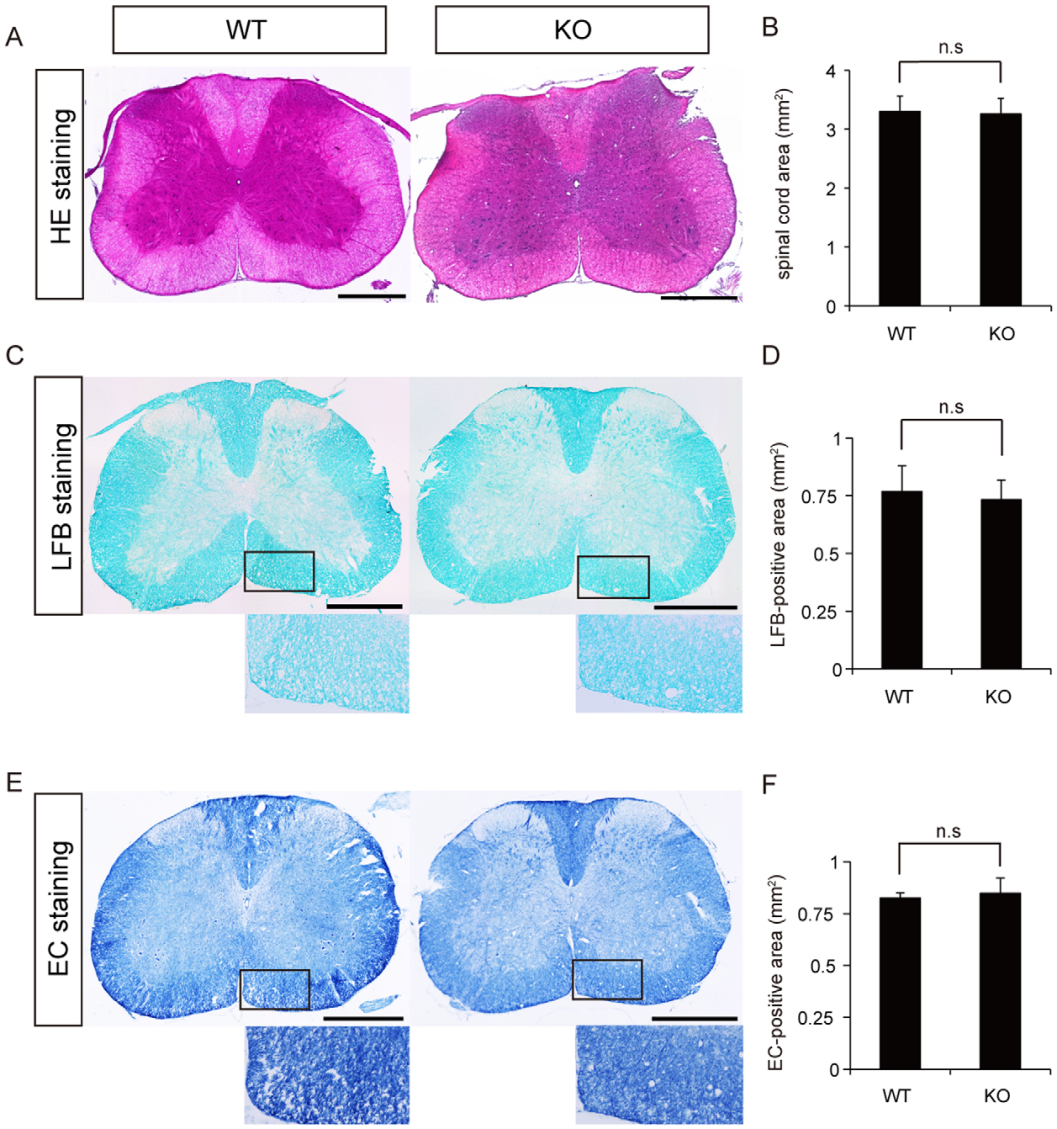
Histological analyses of the spinal cords of WT and CST-KO mice. (A) Representative images of HE-stained axial spinal cord sections. (B) Quantitative analysis of the spinal cord areas of the HE-stained axial sections revealed no significant difference between WT and CST-KO mice. (C) Representative images of LFB-stained axial spinal cord sections. (D) Quantitative analysis of the ventral spinal cord areas measured in the LFB-stained axial sections revealed no significant difference between WT and CST-KO mice. (E) Representative EC-stained images of axially sectioned spinal cords. (F) Quantitative analysis of the ventral spinal cord areas measured in the EC-stained axial sections revealed no significant difference between WT and CST-KO mice. Scale bars: 500 µm in (A), (C), and (E). (B, D, F) Values show the means ± s.d. (n = 4), and significant differences were determined by the Mann-Whitney test.

Histological analyses of the anatomical spinal cord structure in WT and CST-KO mice were performed using HE, LFB, and EC staining. In these analyses, the spinal cords of WT and CST-KO mice were identical in appearance (Fig. 1A, C, E). The transverse area of the axial spinal cord sections did not differ significantly between the two groups (Fig. 1B). LFB and EC staining did not reveal any significant differences between WT and CST-KO mice in the myelinated area of the ventral side (450×250 µm^2^) (Fig. 1D, F).

### MRI and DTI analyses of the WT and CST-KO spinal cords

**Figure 2:**
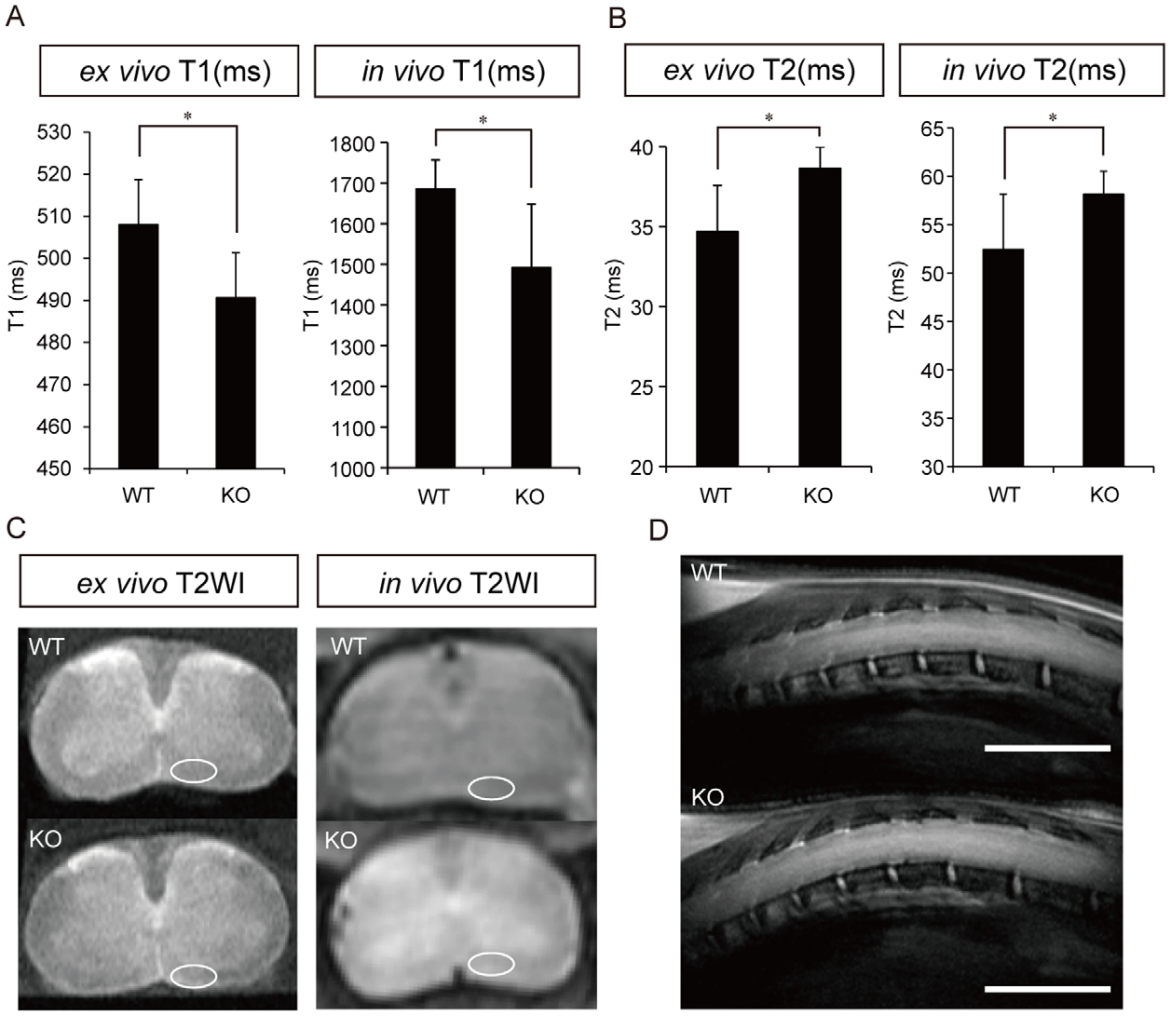
Magnetic resonance images of the spinal cords of WT and CST-KO mice. (A) T1 measurement. The T1 time in the major white matter tracts within the ventral regions was significantly lower in CST-KO mice than in WT mice (circled in panel C), both *ex* and *in vivo*. (B) T2 measurement. The T2 time was significantly higher in CST-KO mice than in WT mice, both *ex* and *in vivo*. (C) Representative axial images of *ex vivo* and *in vivo* T2WI in WT and CST-KO mice. (D) Representative sagittal images of *in vivo* T2WI in WT and CST-KO mice. Scale bars: 5 mm. (A, B) Values shown are means ± s.d. (*ex vivo*: n = 6, *in vivo*: n = 6), with statistical significance determined by the unpaired t-test. *: p<0.05.

To determine whether MRI could detect anatomical structural differences between the spinal cords of WT and CST-KO mice, we obtained high-resolution *in vivo* and *ex vivo* MR images using a cryogenic coil (Fig. 2). The ROIs used to define the ventral side of the spinal cord were drawn on each T1 and T2 map. The *ex vivo* and *in vivo* T1 times for the spinal cord were significantly lower in CST-KO mice than in WT mice (Fig. 2A). On the other hand, the *ex vivo* and *in vivo* T2 times were significantly higher in CST-KO mice than in WT mice (Fig. 2B).

**Figure 3:**
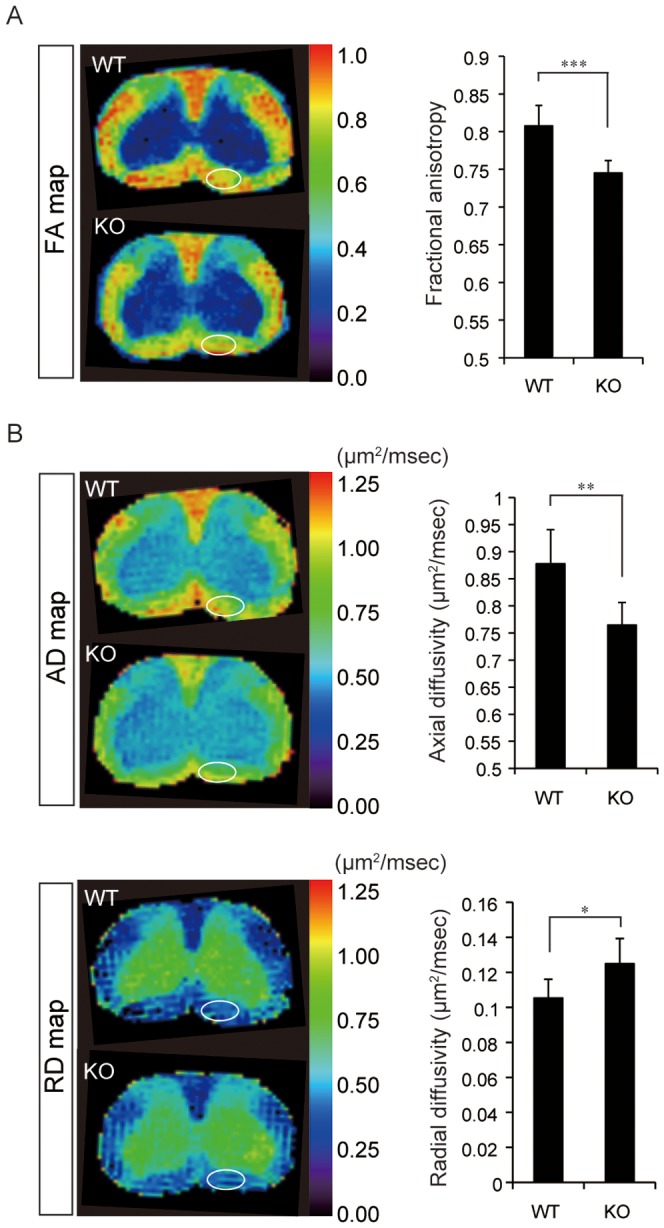
Diffusion tensor imaging in the spinal cords of WT and CST-KO mice. (A) FA map and ROI analysis of the FA values. The FA value of the CST-KO mice was significantly lower than that of WT mice. (B) Axial and radial diffusivity (AD and RD) maps and ROI analyses of the diffusivities. The AD of CST-KO mice was significantly lower than that of WT mice, whereas the RD was significantly higher. (A, B) Values shown are means ± s.d. (*ex vivo*: n = 7), with statistical significance determined by the unpaired t-test. ***: p<0.001, **: p<0.01, *: p<0.05.

After generating DTI maps (e.g., FA, AD, and RD) of the spinal cord in WT and CST-KO mice, the FA values at the ventral side of the spinal cord were measured to analyze DTI differences between WT and CST-KO mice. The FA values were significantly lower in CST-KO than in WT mice (Fig. 3A). The AD values (diffusivity parallel to the long axis of the spinal cord) (Fig. 3B) were also significantly lower in CST-KO than in WT mice. The RD values (diffusivity perpendicular to the long axis of the spinal cord) (Fig. 3B) were significantly higher in CST-KO than in WT mice.

### Histological analyses of spinal cord paranodal junctions in WT and CST-KO mice

**Figure 4:**
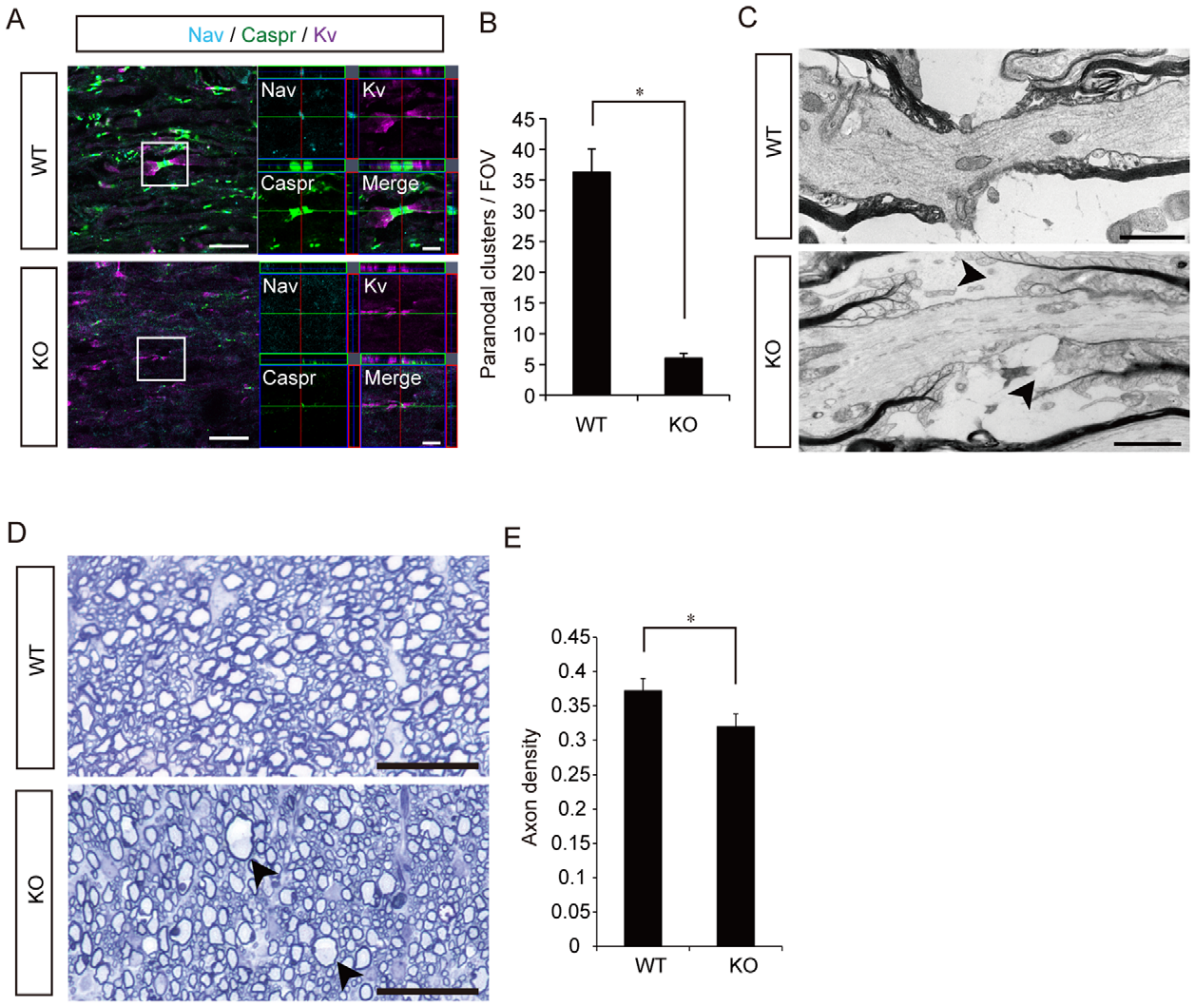
Histological analysis of the paranodal junctions and axons in the spinal cords of WT and CST-KO mice. (A) Representative images of the axons in sagittally sectioned spinal cords stained for Na^+^ channel, Caspr, and K^+^ channel. Scale bar: 20 µm; enlarged view: 5 µm. (B) Quantitative analysis of the number of paranodal clusters per field of view. (C) Ultrastructure of the paranodal junction at a node of Ranvier in a WT and a CST-KO mouse, shown by electron microscopy (arrowheads). Scale bar: 1 µm. (D) Representative toluidine blue-stained images of axially sectioned spinal cord axons. Focal axonal swelling was conspicuous in the white matter of the CST-KO spinal cord (arrows). Scale bar: 50 µm. (E) Quantitative analysis of the axon density. (B, E) Values show the means ± s.d. (n = 4), and significant differences were determined by the Mann-Whitney test. *: p<0.05.

To examine the functional paranodal structure in CST-KO mice, we analyzed the distribution of Nav channels, Caspr clusters, and Kv channels in the spinal cord by paranodal immunostaining [3], [4]. In WT axons, Nav channels were localized to the nodes of Ranvier, Caspr clusters to the paranodal junctions, and Kv channels to the juxtaparanodal regions. In the CST-KO axons, the localization and structure of the Nav channels and Caspr clusters, but not of the Kv channels, were altered (Fig. 4A). The number of paranodal structures per field of view (FOV) (1 FOV = 100×100 µm^2^) was significantly lower in the CST-KO mice than in WT mice (Fig. 4B). Furthermore, electron microscopic examination revealed that the paranodal loops in the CST-KO mice were turned away from the axon (Fig. 4C). Toluidine blue staining showed conspicuous focal axonal swelling (axonal spheroid formation), due to paranodal junction failure in the CST-KO spinal cord (Fig. 4D; arrows). In addition, the axon density was significantly lower in the CST-KO mice than in the WT mice (Fig. 4E).

### Functional and electrophysiological analyses of WT and CST-KO mice

**Figure 5:**
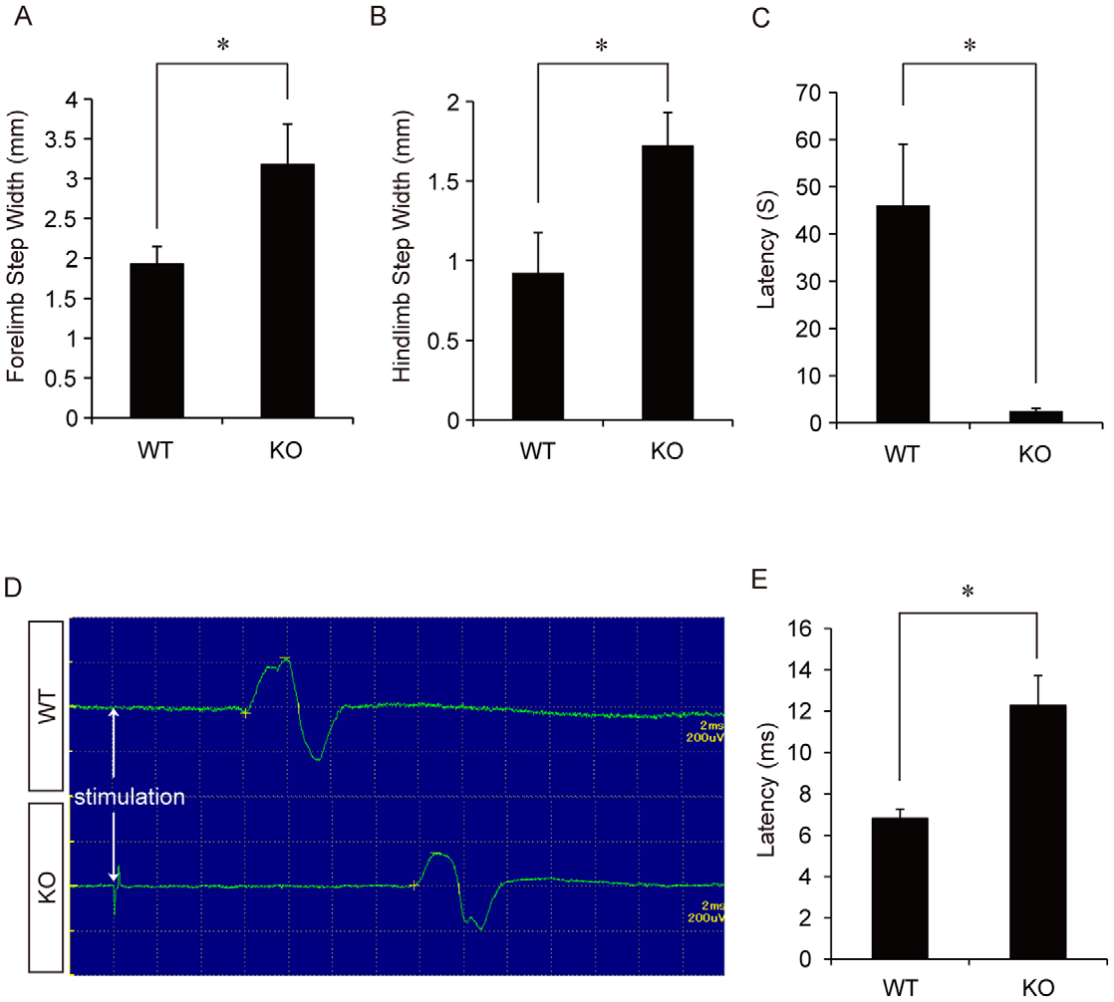
Functional and electrophysiological analyses of WT and CST-KO mice. (A) Forelimb step width of WT and CST-KO mice, obtained by gait analysis. The CST-KO forelimb steps were significantly wider than those of WT mice. (B) Hindlimb step width of each group, obtained by gait analysis. The CST-KO hindlimb steps were significantly wider than those of WT mice. (C) Time on the rotating rod in each group. CST-KO mice stayed on the rod for a significantly shorter time than WT mice. (D) Representative profiles of motor-evoked potentials (MEPs) from each mouse. (E) Quantitative analysis of MEP latency. The MEP latency was significantly longer in CST-KO mice than in WT mice. (A, B, C, E) Values show the means ± s.d. (n = 4), and significant differences were determined by the Mann-Whitney test. *: p<0.05.

Since CST-KO mice show pronounced tremor and progressive ataxia [3], we analyzed their motor function. Footprint analysis with the DigiGait Image Analysis System showed that the steps of CST-KO mice, both with the forelimbs and hindlimbs, were significantly wider than those of WT mice (Fig. 5A, B). In the Rotarod treadmill test, CST-KO mice walked on the rod for significantly less time than did WT mice (Fig. 5C). MEP analysis of the spinal nerve conduction showed that the latency was significantly longer in CST-KO mice than in WT mice (Fig. 5 D, E).

## Discussion

In this study, high-resolution MRI and DTI were able to detect paranodal junction failure in CST-KO mice. To the best of our knowledge, this is the first report of MRI findings for paranodal failure.

Although Bonny et al reported *in vivo* diffusion-weighted images of the mouse spinal cord several years ago [23], it is still difficult to obtain clear images of the spinal cord due to the small size of the mouse and its respiratory and cardiac movements. Cowin et al used *ex vivo* MRI to show subtle anatomical spinal cord changes in transgenic mice [24]. In the present study, use of the CryoProbe, an innovative MRI probe, enabled high-resolution *in vivo* MRI of the spinal cord in WT and CST-KO mice. Both *in vivo* and *ex vivo* MRI of the spinal cord revealed decreased T1 times and increased T2 times in CST-KO mice as compared to WT mice. Consistent with these findings, previous reports have indicated that decreased T1 time and increased T2 time are related to myelination disorders [25], [26]. These results support the utility of *in vivo* MRI with the Cryoprobe to detect the subtle pathological changes present in white matter disorders.

Although DTI effectively reflects histological findings, *in vivo* DTI is difficult to perform due to the lengthiness of the procedure; therefore, we also performed *ex vivo* DTI. We found low AD and high RD in the CST-KO spinal cord. Micro-histological examination detected differences in the axonal and myelinated structures between CST-KO and WT mice. Although Song et al reported that AD and RD are markers of axonal and myelin degeneration, respectively [9], [27], [28], we found that paranodal junction failure resulted in simultaneous diffusivity changes in both parameters. Our findings of low AD and high RD might reflect water movement related to the paranodal junction failure present in CST-KO mice.

Our MRI and DTI findings suggested that the movement of free water within the spinal cord may be more expansive in CST-KO than in WT mice. Although the area of the node of Ranvier makes up about 5% of the whole axon [29], our immunostaining suggested that degeneration occurred in almost the entire node of Ranvier in CST-KO mice. Therefore, the structural degeneration seen in the node of Ranvier might be related to the increase in free water movement and the decrease in anisotropy. Our diffusivity findings might reflect a more subtle, complicated difference in water movement due to paranodal junction failure, suggesting that DTI may be sufficiently sensitive for phenotyping various spinal cord pathologies.

In our histological analyses, HE, LFB, and EC staining did not indicate significant differences between WT and CST-KO mice. However, the subtle paranodal junction failure could be detected by Nav-Caspr-Kv immunostaining, toluidine blue staining, and electron microscopy. Ishibashi et al reported that although 8-week-old CST-KO mice have clinical phenotypes such as ataxia and gait disturbance, compact myelin (internode) destruction is not seen at this age [4]. These findings are compatible with our histological results, which detected subtle paranodal changes but no prominent myelination changes in young CST-KO mice. Because of the paranodal junction failure, the axon density was also significantly lower in the CST-KO mice; these histological findings correlated with the lower FA [15], [27]. Although paranodal junction failure might have caused confounding factors (axonal swelling, axonal degeneration, demyelination) in these MRI findings, these factors should be minimal at this age.

Our behavioral analyses revealed ataxia and gait disturbance in CST-KO mice. The CST-KO mice walked with splayed limbs and could not walk on a slow treadmill. This disability was in agreement with previous reports of paranodal junction failure [3], [30], [31]. Although the MEP latency was significantly longer in CST-KO mice than in WT mice, these electrophysiological findings were inconsistent with a previous analysis [3]. This discrepancy may be explained by differences in the experimental paradigm: Honke et al analyzed mainly peripheral nerve conductivity, and several reports have indicated that the paranodal structure is markedly disorganized in the CNS but only modestly in the PNS in CST-KO mice [32], [33]. Thus, the inconsistency may result from differences in paranodal electrophysiological function between the CNS and PNS.

We found the CST-KO mouse to be a valuable model for studying pathological substrates of paranodal disorders using high-resolution MRI and DTI. CST-KO mice have phenotypes consisting of gait disturbance, ataxia, and electrophysiological deficits, but only subtle histological changes can be detected. Such subtle histological changes may easily have been overlooked in previous MRI studies. Furthermore, in clinical settings, it would probably be difficult to obtain the desired level of resolution, because of the patient's respiratory and cardiac motion and the distortion artifact caused by using echo-planar imaging for rapid acquisition, all of which can significantly decrease the sensitivity of this approach. Combining specific histological methodologies with a newly developed DTI sequence, like SNAILS-DTI [34], should enable the use of high-resolution imaging in the clinic, and may further elucidate agnogenic neurodegenerative diseases.

In conclusion, our findings support the use of high-resolution MRI and DTI as effective new imaging modalities for patients with white matter disorders. In this study, the subtle neurological deficits that resulted from paranodal failure were visible only in micro-histological analyses, yet could be quantitatively analyzed by measuring the T1 and T2 times and DTI parameters. The further development of measurements sensitive to the substructure and composition of white matter will increase our ability to characterize the morphology and state of white matter pathologies. In a clinical setting, such parameters could be useful for diagnosing and understanding the pathologies of progressive myelin diseases such as multiple sclerosis and leukodystrophy.
